# Reduction of Alcoholic Strength: Does It Matter for Public Health?

**DOI:** 10.3390/nu15040910

**Published:** 2023-02-11

**Authors:** Jürgen Rehm, Pol Rovira, Jakob Manthey, Peter Anderson

**Affiliations:** 1 Institute for Mental Health Policy Research, Centre for Addiction and Mental Health, 33 Ursula Franklin Street, Toronto, ON M5S 2S1, Canada; 2 Institute of Clinical Psychology and Psychotherapy, Technische Universität Dresden, Chemnitzer Str. 46, 01187 Dresden, Germany; 3 Centre for Interdisciplinary Addiction Research (ZIS), Department of Psychiatry and Psychotherapy, University Medical Center Hamburg-Eppendorf (UKE), Martinistraße 52, 20246 Hamburg, Germany; 4 Dalla Lana School of Public Health, University of Toronto, 155 College Street, Toronto, ON M5T 1P8, Canada; 5 Faculty of Medicine, Institute of Medical Science, University of Toronto, Medical Sciences Building, 1 King’s College Circle, Toronto, ON M5S 1A8, Canada; 6 Campbell Family Mental Health Research Institute, Centre for Addiction and Mental Health, 33 Russell Street, Toronto, ON M5S 3M1, Canada; 7 Department of Psychiatry, University of Toronto, 250 College Street, Toronto, ON M5T 1R8, Canada; 8 Program on Substance Abuse, Public Health Agency of Catalonia/Agència de Salut Pública de Catalunya, Departament de Salut, Generalitat de Catalunya, Roc Boronat 81-95, 08005 Barcelona, Spain; 9 Department of Psychiatry, Medical Faculty, University of Leipzig, Semmelweisstraße 10, 04103 Leipzig, Germany; 10 Care and Public Health Research Institute, Maastricht University, 6200 MD Maastricht, The Netherlands

**Keywords:** alcohol, alcoholic strength, reduction, no- and low-alcohol beverages, statistical modelling, taxation, mortality, public health

## Abstract

In this work, reduction of alcoholic strength was discussed as a means to reduce consumption and alcohol-attributable harm. Statistical modelling was conducted to (1) estimate its potential for the largest six Western and Central European countries (France, Germany, Italy, Poland, Spain, UK); (2) calculate the increase in taxation necessary to reach this potential, and (3) estimate the mortality gains achieved with the introduction of no- or low-alcohol beverages in the UK and Spain. The high public health potential of reducing alcoholic strength was demonstrated via modelling a scenario in which the strength of all beverages was reduced by 10%, which would avert thousands of deaths in these six European countries per year. However, methods by which to achieve these gains were not clear, as the alcohol industry has shown no inclination toward reductions in the alcoholic strength of beer, wine, or spirits via a reformulation on a large scale. The increase of excise taxation to achieve the public health gains of such a reduction would result in markedly increasing prices—a situation unlikely to be implemented in Europe. Finally, the introduction of beer and wine with an alcoholic strength below 0.5% led to some substitutions of higher-strength beverages, but did not show a marked public health impact. New taxation initiatives to achieve the potential of a reduction of alcoholic strength will need to be implemented.

## 1. Introduction

Alcoholic beverages have different strengths, and one way to reduce intake of ethanol (pure alcohol) in the general population is to substitute higher-strength beverages with lower-strength ones [1]. Three potential pathways to achieve this goal have been distinguished. First, a beverage type with higher alcoholic strength (e.g., spirits with 40% alcoholic strength) can be replaced or partly replaced by lower alcoholic strength beverages (e.g., by beer or wine). One important example for this pathway can be seen in Russia, where the partial substitution of vodka with beer has often been credited as being one of the main drivers of the overall reduction in the level of alcohol consumption and attributable harm [2,3]. This substitution tactic was part of Russia’s national attempt to reduce alcohol abuse and alcohol dependence in the population from 2010 to 2020 [4].

The second and third pathways, which are addressed in detail in this contribution, concern the reformulation of products with lower alcoholic strength, and the introduction of products with low or no alcohol content. To date, reformulation has mainly been done for beer (e.g., a beer with 4.8% alcoholic strength reduced to 4.5% [5]). The brand name has also occasionally been changed, i.e., the new beer has been given a different name. The third pathway concerns the introduction of products with low or no alcohol content, where the term low-alcohol beverage refers to a beverage with an alcoholic strength by volume (ABV) of between 0.05% and 1.2%, and the term no-alcohol beverage refers to beverages with an ABV below 0.05% [6,7]. This market is currently dominated by beer and wine products, but there have also been mixed drinks, liqueurs, and spirits produced with similar alcoholic strengths [7]. Specifically, we examined the public health consequences of these pathways, operationalized via postponed deaths (averted deaths in a year). 

We began with a scenario which assumed that the alcoholic strength of all beverages in six large Western and Central European countries (France, Germany, Italy, Poland, Spain, and the UK) were reduced by 10%. This provided a rough estimate of the potential for reduction in alcoholic strength. More concretely, a 10% reduction would translate, in most countries, to a standard beer with an average of ~5%to ~4.5%, wine from 12.5% to 11.25%, and spirits from 40% to 36% (standard values from WHO, see [8]). While actual average values varied from country to country, using a beverage-specific 10% reduction of the current adult (age 15 and older) alcohol per capita consumption (APC) for each country allowed us to take country variations into account, as APC is based on ethanol (pure alcohol) (see [9]). A 10% reduction in alcoholic strength was chosen because there was good experimental evidence that changes of this magnitude would likely go undetected by consumers, and there seemed to be no titration effect ([10,11]; for a smaller scale analysis on the effects of reformulated products in the UK, see [12]). 

By the end of 2022, the potential of using substitution as a tactic to reduce consumption had not been reached because of market forces or a lack of pledges made by the alcohol industry [12]. As such, the following analyses focused on the potential tax increases necessary to achieve this 10% reduction in alcoholic strength for all alcoholic beverages. These analyses were undertaken using the UK and Spain as examples, as we had the most detailed data on lower strength products for them.

The last analysis was based on actual changes in purchases of no-alcohol beers and wines (ABV < 0.5%) which occurred in Great Britain and Spain (for details, see Appendix B), two countries/regions where this market segment has been very important [13]. We modeled the public health impact of averted deaths during a 6- or 5-year period, respectively, assuming that changes in Great Britain could be extended to the UK as a whole.

To summarize, this paper aimed to:

(1) outline the public health potential of reducing strength of alcoholic beverages in 6 Western European countries (France, Germany, Italy, Poland, Spain, UK);
(2) indicate the taxation changes necessary to achieve this potential in Spain and the UK;
(3) analyze the real changes following the introduction of no-alcohol beers and wines in Spain and the UK.

## 2. Materials and Methods

In the following, we present the methodology employed for the three objectives discussed above (i.e., the mortality reduction associated with a 10% reduction in alcohol strength leading to a 10% reduction in adult (age 15 and older) alcohol per capita consumption (APC) for beer, wine, and spirits; the increase in excise taxation necessary to obtain this 10% reduction; and the mortality reduction associated with the introduction of non-alcoholic beer and wine in the UK and Spain).

### 2.1. Causes of Death Attributable to Alcohol Use

We based our selection of causes of death which were fully or partially attributable to alcohol use on the overview of Rehm and colleagues [14]. Mortality data, i.e., number of deaths by cause, sex, and age groups, were obtained from the website of the Global Burden of Disease study [15]. For liver cirrhosis, following the tradition of *The Global Status Reports on Alcohol and Health* [8], we estimated the proportion attributable to alcohol by attributable fraction methodology (see below). The International Classification of Diseases, 10th revision (ICD-10) codes for all causes of death attributable to alcohol consumption can be found in Shield et al. [16].

### 2.2. Exposure

For our first aim, for all six countries, we used adult alcohol per capita consumption data from the World Health Organization [17]. For the distribution of overall alcohol consumed by sex and age, the data were obtained from Manthey and colleagues [18].

The differences in the number of deaths, given changes in consumption levels, were obtained by taking the difference between the deaths calculated using the current APC and the deaths derived using the modified APC. For the case of the 10% reduction for Germany, France, Italy, Poland, Spain, and the UK, the new APC value was calculated to be the equivalent of 90% of the APC for 2019.

For our third aim—modeling the reduction of APC associated with the introduction of non-alcoholic beer and wine—the procedure was the same. However, while we already knew the overall sex- and age-distribution for the consumption of alcohol, the specific distribution for drinking beer and wine was unknown.

We therefore applied Formula (1), where, for example, APC_beer_ is the contribution to the total APC from beer consumption and %B is the percentage of APC due to beer. Only these three beverages were considered, and the sum of their percentages was set to equal 1.

[Formula 1]

(1) $$APC=APC_{beer}+APC_{wine}+APC_{spirits}=APC*\left(\%B+\%W+\%S\right)$$

Splitting the APC into the contributions from each beverage type—and knowing how each one decreased—allowed us to calculate the resulting APC. 

The specific exposure data on the impact of introducing non-alcoholic beer and wine in Great Britain and Spain were obtained from the work of Anderson and colleagues (for details, see Appendix B and Table A3).

Finally, consistent with the procedures for the comparative risk assessment by the WHO [8], all effects were assumed to occur in the same year, with the exception of cancer, where a 10-year gap for effects from cancers was assumed due to a lag effect [19] and therefore the exposure rate for the 10 years prior was used. We assumed that the APC, the percentage of current drinkers, and the percentage of heavy episodic drinkers decreased by the same proportions.

### 2.3. Calculating the Number of Deaths Averted in 2019

Deaths averted due to changes in APC were estimated by applying alcohol-attributable fractions (AAF) methodology [20,21]. For partially alcohol-attributable diseases, we estimated the alcohol-attributable mortality for the year 2019, and then repeated the same estimates with the assumptions based on the different exposure scenarios. For fully alcohol-attributable disease categories (alcohol use disorders; alcoholic cardiomyopathy), the methodology proposed by Churchill et al. [22] was used. 

The formula for the AAF is shown in Formula (2) where P_i_ is the prevalence for a particular consumption group i, and RR_i_ is its relative risk (the relative risks were taken from Shield et al. [16]), the groups considered are the abstainers (*abs*), the former drinkers (*FD*), and the current drinkers (*CD*). 

[Formula 2]

(2) $$AAF=\frac{P_{abs}RR_{abs}+P_{FD}RR_{FD}+\int_{0}^{150}P_{CD}\left(x\right)RR_{CD}\left(x\right)dx-1\;}{P_{abs}RR_{abs}+P_{FD}RR_{FD}+\int_{0}^{150}P_{CD}\left(x\right)RR_{CD}\left(x\right)dx}$$

By multiplying the total number of deaths for the respective causes by the AAFs obtained, the deaths attributable to alcohol were determined.

For totally attributable diseases, the AAF methodology from the formula above could not be used because AAFs are 100% alcohol-attributable by definition. In this case, Formula (3) below was used, where N is the number of deaths, S is the total population number, dF(x;μ) is the gamma distribution for a mean consumption μ, and p(x;k,t) is the function developed in Formula (4). In Formula (4), x is the alcohol consumption, *t* is the threshold for heavy drinking—40 g/day for women and 60 g/day for men—and k is an unknown parameter that fits Formula (3).

[Formula 3]

(3) $$\int_{0}^{150}p\left(x;k,t\right)dF\left(x;\mu \right)=\frac{N}{S}$$

[Formula 4]

(4) $$p\left(x;k,t\right)=\{\begin{array}{c} 0\;x<t\\ \exp\left(k\left(x-t\right)\right)-1\;x\geq t \end{array}$$

For this calculation, the only unknown parameter in Formula (3) is *k*. To obtain the value that fit for our cases, the Newton–Raphson method was applied. Once this value was obtained, it was used in the modified scenario with the reduced consumption to find the resulting number of deaths. 

### 2.4. Required Taxation

A decrease in alcohol consumption could be achieved by increasing the taxes on alcoholic beverages and, consequently, their prices. The relationship between both changes would be dependent on the price elasticity (see Formula (5), where %∆Q is the change in the percentage of consumption, Q_i_ is the consumption before this change, Q_f_ is the consumption after this change, and %∆P is the increase in the percentage of price).

[Formula 5]

(5) $$E=\frac{\%∆Q}{\%∆P}=>\%∆P=\frac{\%∆Q}{E}=\frac{Q_{i}-Q_{f}}{Q_{i}}\frac{1}{E}$$

The literature showed that price elasticity on alcoholic beverages did not differ widely across different countries [23,24,25,26,27]; it was mostly differentiated between alcoholic beverages and their degree of popularity. This was because the most preferred alcoholic product had elasticities closer to 0 than those that were not as widely appreciated. For the most preferred beverage type in each country, −0.36 (95% CI: −0.48, −0.24) was used. Likewise, −1.2 (95% CI: −1.44, −0.96) was use for the least preferred alcoholic beverage and, since three beverage types were considered (beer, wine, and spirits), −0.6 (95% CI: −0.72, −0.48) was used for the third [23,28]. Additionally, heavy drinkers had lower elasticities due to their stronger dependence on these beverages (often without a clear preference for a particular beverage type). For this reason, the value elasticity we used was −0.28 (95% CI: −0.37, −0.19) [27]. No cross-elasticities were considered, as the current scenarios implied the same proportional reductions for each alcohol beverage type.

As noted above, if the %∆Q desired was known, the %∆P that must be applied could also be calculated. Moreover, the total price of the alcoholic beverages was considered as the sum of the production price and the taxes. However, since we knew the percentage of taxes in the final price, the prices could be expressed in an alternative way, as provided in Formula (6). Then, the definition of %∆P could be found in Formula (7):

[Formula 6]

(6) $$P_{T}=P_{Prod}+P_{Taxes}=\frac{P_{Prod}}{\left(1-\%P_{Taxes}\right)}$$

[Formula 7]

(7) $$\%∆P=\frac{P_{i}-P_{f}}{P_{i}}=\frac{P_{i}-P_{i}\left(\frac{1-\%tax}{1-\%tax^{\prime }}\right)}{P_{i}}=1-\left(\frac{1-\%tax}{1-\%tax^{\prime }}\right)$$

where P_i_ is the price before the change, P_f_ is the price after the change, %tax is the percentage of the total price due to taxes, and %tax’ is the percentage of the total price required to generate the amount of tax required to achieve the desired %∆Q. Thus, by combining Formulas (5) and (7), the value of this %tax’ could be found (see Formula (8)).

[Formula 8]

(8) $$\%tax^{\prime }=1-\frac{1-\%tax}{1-\frac{\%∆Q}{E}}$$

All of the above depended on the assumption that there was a full pass-through between taxation and price, i.e., that the alcohol producers and sellers pass on the higher taxes fully to the purchaser in the final price [29].

The data on current alcoholic beverage prices and their taxation levels were extracted from Neufeld et al., 2022 [30].

### 2.5. Required Taxation

We did a sensitivity analysis, modeling the effects of a 5% reduction of alcoholic strength and the required taxation. 

## 3. Results

### 3.1. The Public Health Potential of Substitution

Table 1 gives the main results on the scenario of a 10% reduction in alcoholic strength for all three beverage types in the countries selected. Depending on the drinking level, the size of the country, sex, and sex-specific structure of the causes of death, between 5.01% and 10.25% of all alcohol-attributable deaths could have been averted in 2019 (i.e., the deaths would happen in subsequent years). This corresponded to between 0.42% (women in Spain) and 1.26% of all deaths in the country (Polish men). 

The absolute numbers were, of course, markedly impacted by the size of the country. Thus, Germany, as expected, gained the most from a 10% reduction of beverage strength, with a total of 4517 deaths averted in one year. For all countries, as expected, given the sex differences in alcohol consumption [18], more deaths would be averted in men than in women. This ratio was highest in Italy and Poland.

Table A1 in Appendix A gives the distribution of deaths by cause. For women in all countries, cardiovascular disease deaths comprised the largest category, followed by a category denoted as “other disease” (primarily comprising alcohol use disorders). The situation was more complex for men, where cancer deaths constituted the largest category in four of the countries (France, Italy, Spain, and the UK), while “other disease” (again, mainly alcohol use disorders) was the category with the highest number of deaths in Poland, and cardiovascular disease was the highest in Germany. Cancer was modeled based on exposure rates from 2009. As expected, the four biggest categories (cancer, cardiovascular disease, digestive disease (mainly liver cirrhosis), and injury) made up the vast majority of alcohol-attributable deaths averted. However, the number of deaths averted due to the category of “other disease”, which included alcohol use disorders, was surprisingly high (see Table A1).

### 3.2. Taxation Required to Achieve a 10% Reduction in Alcoholic Beverage Strength

With respect to the taxation necessary to achieve this effect in Spain and the UK, we first displayed the current level of taxation by providing the tax share (Table 2a, based on [30]). Tax share denoted the proportion of tax included in the final price of the alcoholic beverage.

Table 2 gives the current tax share (Table 2a) and tax share necessary to achieve the effect of a 10% reduction in alcoholic strength (Table 2b), along with the resulting increase in price (Table 2c)

Table 2a shows that alcohol excise taxes in both countries were low, although the UK showed markedly higher tax shares. In Spain, there was no tax at all on wine, and, for beer, excise taxes made up less than 3% of the final price. Given these low tax shares and the fact that alcoholic beverages were relatively inelastic [23,27], especially for preferred beverage types and heavy drinkers (see Methodology section above), marked increases were necessary (see Table 2b), and the resulting price increases were considerable (see Table 2c).

### 3.3. Public Health Evaluation of the Introduction of Low/No Alcoholic Beverages

For the aforementioned evaluations, we only dealt with hypothetical scenarios. The final evaluation was based on the real-life experience of two countries which previously introduced no-alcohol beers and wines (ABV < 0.5%), using data from Great Britain to estimate effects for the whole UK (six years) and for Spain (five years). Table 3 summarizes the results for all years combined.

For both countries, the introduction of beer and wine with an alcoholic strength of less than 0.5% led to a small substitution effect, which, while possibly statistically significant, was not relevant to public health (see Table 3; Table A2 in Appendix A gives the distribution of deaths by cause). The largest effect was seen for men in the UK, where the number of deaths averted per year amounted to 16.

### 3.4. Sensitivity Analyses

The sensitivity analyses (Appendix C; Table A4, Table A5 and Table A6) showed that, even for a reduction of alcoholic strength of 5%, the public health gains were substantial, with associated price increases between 16% and 36%.

## 4. Discussion

This study had three aims: to estimate the public health potential of reducing the strength of alcoholic beverages in six Western and Central European countries, to indicate the taxation changes necessary to achieve this potential, and to analyze the real changes that followed the introduction of no-alcohol beers and wines in Spain and the UK.

Reduction of alcoholic strength clearly demonstrated public health potential. An examination of a scenario in which the strengths of all beverages were reduced by 10% showed the potential for preventing thousands of deaths in six European countries. Even a reduction in strength of alcoholic beverages by 5% resulted in substantial mortality gains. However, the way to achieve these gains remained unclear, as the alcohol industry has shown no inclination toward reducing the alcoholic strength of beer, wine, or spirits via a reformulation on a large scale. Introducing taxation to achieve the public health gains of such a reduction would result in markedly increasing prices—a situation which is unlikely to occur in Europe, where alcohol has traditionally been very affordable [30], and where even adjustments for inflation have rarely been made [31]. We discussed potential alternatives below. Finally, the introduction of beer and wine with alcoholic strengths of less than 0.5% in two countries led to some substitution of higher-strength beverages but did not have a marked public health impact.

Prior to further discussion, we felt it important to point out some potential limitations of this work. Regarding the potential public health impact of a reduction of the alcoholic strength of all beverages, more empirical evidence is needed at the population level. We would need to examine more examples of reductions based on reformulations of existing alcoholic beverages, such as was done in the UK [12]; however, most of the effects involved reformulated beer, and in the UK, a single case study accounted for most of the effect. Clearly, more such natural experiments would be necessary for different countries. With respect to the taxation models, the taxation increases necessary to effect such a change could be overestimated in our model even though we used standard elasticities. Recent analyses (e.g., [32]) showed marked underestimation of the beneficial effects of taxation using the standard elasticities from meta-analyses conducted a decade ago [23,24,25,27] when compared with direct estimates of interrupted time-series analyses. However, more recent meta-analyses showed similar values [33]. Finally, all models were based on relative risks derived from meta-analyses for dose–response relationships [34], and these could pose challenges when applied to reductions in drinking and its consequences.

Assuming that 1) the reduction of alcoholic strength in alcoholic beverages shows great potential, 2) the alcohol industry shows no inclination toward reformulating their products, and that 3) huge increases in excise taxation are improbable, one possible solution might be to use different taxation strategies. For example, Corfe [35] suggested a fixed duty per gram of alcohol, which would be multiplied by the alcoholic strength, but be steeper at lower strengths to incentivize low-strength products. Another potential answer, which was associated with reducing harm in the Northern Territory of Australia, was the implementation of a levy on all beers having an alcoholic strength greater than a threshold value of 3.0% [36,37]. However, as other measures were implemented at the same time, causality could not unequivocally be established. Authorities in countries and supranational organizations such as the EU should test different ways to achieve reductions in alcoholic strength, and how best to implement them. Currently, the knowledge base is not large enough to predict exactly what would happen with different forms of taxation (for general principles, see **[38]**).

Finally, while—against some predictions to the contrary—the introduction of no- and low- alcohol beverages has led to general substitution effects [5], the uptake of such products by the general population has not been large enough to produce sizable public health effects. It would, therefore, be up to the industry to demonstrate that there could be conditions under which a public-health relevant substitution would take place.

## 5. Conclusions

The reduction of alcoholic strength has demonstrated high life-saving potential. As this potential has yet to be realized by the alcohol industry, new taxation initiatives to achieve it need to be implemented.

## Figures and Tables

**Table 1:** Potential averted deaths due to a reduction of alcoholic strength in 2019.

Country	Sex	All Deaths	95% CI		% AA Deaths	95% CI		% of All Deaths	95% CI	
DEU	Women	1781	1414	4097	10.25	6.15	13.04	0.73	0.54	1.54
DEU	Men	2736	2384	3632	6.59	4.64	8.49	1.12	0.98	1.48
ESP	Women	403	282	1376	6.14	4.43	10.39	0.42	0.28	1.32
ESP	Men	804	629	1242	5.35	3.22	6.93	0.87	0.68	1.34
FRA	Women	880	709	2469	9.02	5.7	11.48	0.67	0.48	1.68
FRA	Men	1623	1405	2189	6.09	4.13	7.81	1.19	1.03	1.59
UK	Women	779	632	2289	8.07	5.92	11.31	0.57	0.42	1.52
UK	Men	1276	1055	1853	6.07	4.0	7.7	0.87	0.71	1.26
ITA	Women	480	340	1928	5.01	3.48	11.05	0.28	0.18	1.02
ITA	Men	1053	828	1740	5.88	3.91	7.4	0.70	0.55	1.16
POL	Women	649	474	1580	7.51	4.7	11.52	0.56	0.39	1.31
POL	Men	1557	1363	2073	6.62	4.91	8.51	1.26	1.1	1.68

Country abbreviations: DEU: Germany; ESP: Spain; FRA: France; UK: United Kingdom; ITA: Italy; POL: Poland.

**Table 2:** Current tax share (**a**) and tax share necessary to achieve the effect of a 10% reduction in alcoholic strength, along with (**b**) resulting increase in price (**c**).

(a)		
**% Current tax share**	**UK**	**ESP**
Beer	21.7%	2.8%
Wine	21.8%	0%
Spirits	32.1%	23.8%
**(b)**		
**% Necessary tax share**	**UK**	**ESP**
Beer	54.6%	40.0%
Wine	46.6%	33.4%
Spirits	48.8%	44.8%
**(c)**		
**% Increase in Price**	**UK**	**ESP**
Beer	72.2%	61.9%
Wine	46.4%	50.1%
Spirits	32.6%	38.0%

**Table 3:** Public health impact of introducing beer and wine with less than 0.5% alcoholic strength in the UK (6 years) and Spain (5 years)—cumulative results.

Country	Sex	All Deaths	95% CI		% AA Deaths	95% CI		% of All Deaths	95% CI	
ESP	Women	18	14	51	0.28	0.21	0.42	0.02	0.01	0.05
ESP	Men	36	30	51	0.24	0.15	0.32	0.04	0.03	0.06
UK	Women	62	48	183	0.65	0.45	0.91	0.05	0.03	0.12
UK	Men	94	76	142	0.45	0.29	0.6	0.06	0.05	0.10

## Data Availability

Underlying data on alcohol per capita consumption can be obtained from the WHO at https://www.who.int/data/gho/data/themes/global-information-system-on-alcohol-and-health. (accessed on 10 October 2022).

## Appendix A

**Table A1:** Causes of death averted by a 10% reduction of alcoholic strength in 2019 by country and sex.

Country	Sex	Infectious Diseases	Cancer	CVD	Digestive Diseases	Injuries	Other Diseases	All Diseases
DEU	Women	51	172	800	207	124	427	1781
DEU	Men	123	471	792	249	347	753	2736
ESP	Women	26	59	181	74	39	24	403
ESP	Men	68	255	164	114	132	71	804
FRA	Women	54	118	263	98	115	232	880
FRA	Men	105	360	213	138	333	474	1623
UK	Women	77	123	236	108	60	174	779
UK	Men	165	368	221	130	186	208	1276
ITA	Women	16	53	209	110	65	27	480
ITA	Men	58	270	255	167	217	86	1053
POL	Women	22	66	302	67	54	138	649
POL	Men	65	209	398	116	244	526	1557

**Table A2:** Cumulative causes of death averted by the introduction of beer and wine with less than 0.5% alcoholic strength in Spain and the UK over a 5- and 6-year period, respectively.

Country	Sex	Infectious Diseases	Cancer	CVD	Digestive Diseases	Injuries	Other Diseases	All Diseases
ESP	Women	4	7	25	11	7	5	58
ESP	Men	9	28	23	17	23	12	113
UK	Women	13	20	40	17	11	34	135
UK	Men	26	56	35	20	35	38	209

CVD: cardiovascular diseases.

## Appendix B. Estimation of the % Reduction of Pure Alcohol Associated with No-Alcohol Products in Great Britain and Spain

### Appendix B.1. Methods

Kantar Worldpanel’s (KWP) household shopping panels, previously described [5,12,39,40], were used for Great Britain and Spain: 5.02 million separate alcohol purchases were recorded by 79,415 British households, over six years (2015–2020); and 1.29 million separate alcohol purchases were recorded by 18,954 Spanish households, over five years (from the second quarter of 2017 to the end of the first quarter of 2022).

In each country, households were recruited via stratified sampling, with targets set for jurisdictional area, household size, and age of the main shopper, with the panels being representative of households in either country. Households provided demographic information when joining the panel, followed by annual updates and quality checks. Using barcode scanners, households recorded all alcohol purchases brought into the home from all store types, including Internet shopping. Households were grouped into one of four age groups based on the age of the main shopper: 18–24; 25–44; 45–64; 65+ years.

For each individual purchase, the provided data included the type and volume of the purchase, the brand, and the percent alcohol by volume (ABV). The volume purchased was combined with ABV to calculate grams of alcohol purchased. Beers and wines were separated into groups by ABV as follows: <0.5%; 0.5% to 3.5%; and >3.5%.

Data was first prepared by taking any day that a household bought alcohol, adding up the amount of alcohol purchased in both volume and grams, and then dividing by the number of adults in the household. Then, for each study day of the time-periods (2015–2020 for Great Britain, and the second quarter of 2017 to the end of the first quarter of 2022 for Spain), the mean volume and grams of purchases were calculated for all products and for beers and wines.

To assess the associations between substitution of higher strength products (ABV > 3.5%) with no-alcohol products (ABV < 0.5%) on purchases of grams of alcohol over time, Auto-Regressive Integrated Moving Average (ARIMA) models were used.

The dependent variable was the total number of grams of alcohol purchased per adult per household per day of purchase averaged across all households for each study day. The independent variables were:

- Measure of substitution calculated separately for beers and wines as (the volume (mL) of purchases of all beer/wine with an ABV > 3.5% plus volume (mL) of purchases of no-alcohol beer/wine with an ABV < 0.5%) minus volume (mL) of purchases of no-alcohol beer/wine with an ABV < 0.5%;
- Impact of the event of COVID lockdown in 2020, dummy-coded as 0 before the event and as 1 from the event onward.

Based on visual observation and Q-Q plots, the continuous dependent and independent variables averaged across all households per day of purchase were normally distributed. A time-series modeler function [41] was used to estimate the best-fitting non-seasonal and seasonal ARIMA model that: (a) specifies degrees of differencing and/or a square root or natural log transformation to ensure a stationary series; and, (b) specifies autoregressive and moving average orders, resulting in the following ARIMA non-seasonal and seasonal models (0, 0, 14) (0, 0, 0). The ARIMA models were repeated separately for each age group.

### Appendix B.2. Results

Over the respective full time periods, no-alcohol substitution increased by 37.3 mL per adult per British household and by 54.8 mL per adult per Spanish household, and no-alcohol wine substitution increased by 41.8 mL per adult per British household and by 45.7 mL per adult per Spanish household.

These increases in substitution over the full time periods were associated with decreases over the full time periods in all purchased grams of alcohol in Great Britain due to no-alcohol beer of 1.68 g (a 2.0% reduction of the average of all purchases during the first year of the time period) and due to no-alcohol wine of 3.86 g (4.2% reduction); the respective associations for Spain were for beer 1.84 g (1.95% reduction) and for wine 4.0 g (4.2% reduction).

**Table A3:** Associated reductions or pure alcohol by age of the main household shopper.

Country	Age Group	No-Alcohol Product	% Reduction Pure Alcohol
Great Britain	18–24	Beer	3.34
Great Britain	25–44	Beer	2.22
Great Britain	45–64	Beer	0.91
Great Britain	65+	Beer	1.91
Great Britain	18–24	Wine	4.41
Great Britain	25–44	Wine	5.04
Great Britain	45–64	Wine	2.79
Great Britain	65+	Wine	2.48
Spain	18–24	Beer	4.11
Spain	25–44	Beer	2.30
Spain	45–64	Beer	0.58
Spain	65+	Beer	0.47
Spain	18–24	Wine	5.00
Spain	25–44	Wine	2.47
Spain	45–64	Wine	5.68
Spain	65+	Wine	3.31

## Appendix C. Sensitivity Analysis—5% Alcohol Consumption

**Table A4:** Causes of death averted by a 5% reduction of alcoholic strength in 2019 by country and sex.

Country	Sex	Infectious Diseases	Cancer	CVD	Digestive Diseases	Injuries	Other Diseases	All Diseases
DEU	Women	26	86	408	101	61	221	902
DEU	Men	60	227	392	118	169	368	1334
ESP	Women	13	30	93	37	19	13	204
ESP	Men	33	124	80	54	64	35	390
FRA	Women	27	59	134	48	57	120	445
FRA	Men	51	173	104	66	162	232	788
UK	Women	39	62	122	53	30	90	395
UK	Men	80	178	108	62	91	102	620
ITA	Women	8	27	108	54	32	14	243
ITA	Men	28	133	125	80	106	43	516
POL	Women	11	33	153	33	26	71	328
POL	Men	31	100	213	55	119	256	774

**Table A5:** Averted deaths by a reduction of alcoholic strength in 2019 (5% reduction of alcoholic strength).

Country	Sex	All Deaths	95% CI		% AA Deaths	95% CI		% of All Deaths	95% CI	
DEU	Women	902	690	1987	5.19	2.92	6.65	0.37	0.26	0.76
DEU	Men	1334	1150	1790	3.21	2.25	4.11	0.55	0.47	0.73
ESP	Women	204	131	661	3.1	2.1	5.25	0.21	0.13	0.64
ESP	Men	390	313	594	2.6	1.58	3.44	0.42	0.34	0.65
FRA	Women	445	356	1210	4.56	2.6	5.88	0.34	0.24	0.84
FRA	Men	788	690	1062	2.95	2.04	3.81	0.58	0.5	0.77
UK	Women	395	313	1152	4.09	2.88	5.64	0.29	0.21	0.76
UK	Men	620	507	892	2.95	1.92	3.82	0.42	0.34	0.61
ITA	Women	243	166	926	2.54	1.77	5.47	0.14	0.09	0.51
ITA	Men	516	415	846	2.88	1.94	3.75	0.34	0.28	0.57
POL	Women	328	237	791	3.8	2.32	6.06	0.29	0.19	0.65
POL	Men	774	658	1027	3.29	2.29	4.22	0.63	0.54	0.83

**Table A6:** (**a**) Tax share necessary to achieve the effect of a 5% decrease in consumption. (**b**) Resulting increase in price.

(a)		
**% Necessary tax share**	**UK**	**ESP**
Beer	42.5%	25.8%
Wine	36.5%	20.0%
Spirits	41.6%	36.0%
**(b)**		
**% Increase in Price**	**UK**	**ESP**
Beer	36.1%	31.0%
Wine	23.2%	25.1%
Spirits	16.3%	19.0%
